# A Novel and Efficient In Vitro Organogenesis Approach for *Ajuga lupulina* Maxim

**DOI:** 10.3390/plants10091918

**Published:** 2021-09-15

**Authors:** Qinggui Wu, Honglin Yang, Yulin Yang, Jinyu He, Erga Aer, Yonghong Ma, Lijuan Zou

**Affiliations:** 1 Ecological Security and Protection Key Laboratory of Sichuan Province, Mianyang Normal University, Mianyang 621000, China; qgwu30@163.com (Q.W.); yanghonglin926@gmail.com (H.Y.); aerga589@gmail.com (E.A.); 2 Sichuan Academy of Forestry Sciences, Chengdu 610081, China; yangdoudou926@gmail.com; 3 College of Life Sciences, China West Normal University, Nanchong 637009, China; jjyyhe26@163.com (J.H.); ayuwure58211@gmail.com (Y.M.)

**Keywords:** *Ajuga lupulina*, leaf, embryo-like structures (ELSs), organogenesis

## Abstract

This work was aimed at establishing an effective approach for in vitro propagation of *Ajuga lupulina* Maxim, a medicinal and ornamental plant mainly found in eastern Xizang, in the western Sichuan region of China. We report an optimum response in the proliferation of axillary shoots from nodal segment explants (10.2 shoots/explant) on MS medium containing 3.0 mg L^−1^ of 6-benzyladenine (BA). When BA and TDZ individually or in combination with NAA were employed for adventitious shoot regeneration, shoots and embryo-like structures (ELSs) were noted in the callus from leaf explants. The maximum response of 26.4 shoots /explant (81.6%) and 12.0 ELSs/explant were ascertained on MS medium with 4.0 mg L^−1^ TDZ and 0.1 mg L^−1^ NAA. The leaf despite browning still demonstrated a high regeneration capacity. TDZ (2.0 mg L^−1^) and BA (2.0 mg L^−1^) along with NAA (0.01 mg L^−1^) were found to perform well for shoot regeneration via callus from shoot tip explants. The best for rooting was MS medium (half-strength) containing indole-3-butyric acid (IBA: 1.5 mg L^−1^) and (NAA: 0.5 mg L^−1^) with the maximum number of roots (25.8 per shoot) and the highest rooting frequency (81.71%). The survival of the plantlets in the greenhouse was 78.2% indicative of successful acclimatization. This work is the first report of a consistent, definitive, and unique protocol for *A. lupulina* regeneration, paving the way for the in vitro preservation of such significant genetic resources and also further allied systems based on such callus-based or embryo-based approaches.

## 1. Introduction

The genus *Ajuga* (Lamiaceae) encompasses more than 300 annual and perennial species across the globe [1,2]. The traditional use of several of these species to address diabetes, fever, rheumatism, tuberculosis, and high blood pressure on account of the presence of many bioactive compounds with anti-inflammatory, antioxidant, antibacterial, or anti-tumor activities is known [3,4,5,6]. The use of some *Ajuga* species as ornamentals, given their distinctive leaves, bracts, or flowers, is also prevalent. The use of approximately 18 *Ajuga* species in the Chinese ethnomedical system with some included in the Pharmacopoeia of the People’s Republic of China and other species being employed in traditional Chinese medicine (TCM) for the expulsion of wind, quelling heat, and treating tuberculosis has also been reported [7]. The recent years have seen a spike in attention on *Ajuga* species given their medicinal importance, resulting in a slew of studies on them.

**Figure 1:**
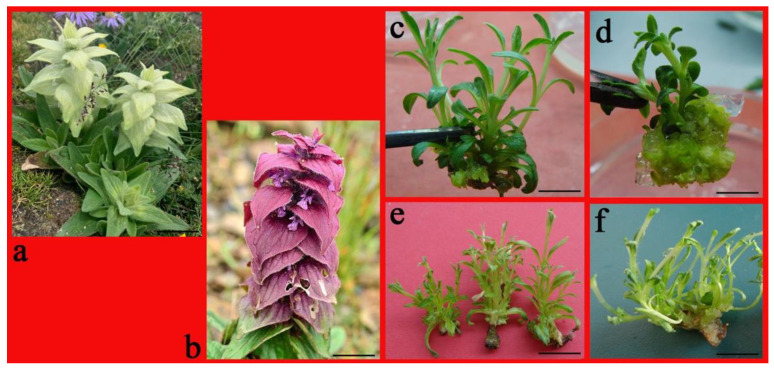
The widely grown *Ajuga lupulina* plants and its axillary shoot proliferation. (**a**) *A. lupulina* with yellowish white bracts. (**b**) *A. lupulina* with purple bracts. (**c**) Axillary shoot proliferation on 2.0-mg L^−1^ BA-containing culture, demonstrating some callus at the base. (**d**) Axillary shoot germination on 2.0-mg L^−1^ TDZ-containing culture with evident callus at the base; (**e**) Axillary shoot proliferation on 2.0-mg L^−1^ ZT-containing culture and. (**f**) 2.0-mg L^−1^ KT-containing culture with little callus and hyperhydric leaves. Bar = 3 cm (**a**,**b**), 1 cm (**e**,**f**), 5 mm (**c**,**d**).

*Ajuga lupulina* Maxim is a perennial herb known for its medicinal properties and ornamental value [7,8]. Its yellowish or purple petals with purple spots assembled beneath the cascading bracts have a unique charm to catch people’s attention (Figure 1a,b). It is mostly distributed in the western region of Sichuan, including Qinghai, Xizang, and Gansu at high altitudes of 1900–3500 m in China. Traditional medicine has used *A. lupulina* whole plants to address sore throat, hypertension, urinary calculi, or epilepsy [7,9]. The main active compounds here are diterpene, lupulin, 6-deacetylajugarin IV, and luteolin to find its unique place in the Chinese Pharmacopoeia [10].

Due to poor seed viability, the predominant propagation of *A. lupulina* is via rhizome division. Yet, this conventional propagation faces issues in the form of slow multiplication [11]. Owing to their medicinal and ornamental aspects, pharmacological and phytochemical research entails the collection of *A. lupulina* plants from their natural sites. The limited distribution of this herb along tough habitats (sandy beaches or steep slope crevices), coupled with over-harvesting, may cause *A. lupulina* to become endangered. This necessitates an alternative and efficient propagation protocol of this plant on a large-scale.

While there are micropropagation protocols for several *Ajuga* species [2,3,11,12,13], a regeneration approach for *A. lupulina* is lacking. Most of the studies focused on the rapid propagation of them, and the literature is scarce regarding the somatic embryogenesis in Ajuga species except for *A. bracteosa* [3]. In the present study, successful in vitro micropropagation of *A. lupulina* is presented. Apart from the varying levels and plant growth regulators (PGRs) combinations utilized, the developmental features of embryo-like structures (ELSs) of *A. lupulina* were also recorded. This work elucidates a dependable and consistent approach for in vitro regeneration via organogenesis (direct and indirect) and the induction of ELSs, opening up the possibility of mass multiplication and germplasm conservation.

## 2. Results

### 2.1. Impact of PGRs on Axillary Shoot Proliferation from Nodal Segments

All cytokinin treatments resulted in the production of axillary shoots; no shoot initiation was observed for the medium lacking PGRs over 35 days of incubation (Table 1). There was a significant difference in the number of axillary shoots produced depending on cytokinin and concentrations, with TDZ producing the lowest number compared to the other cytokinins (Table 1). In the medium supplemented with 6-benzyladenine (BA), one nodal segment proliferated into axillary shoots (7.7–10.2) and produced robust shoots with dark-green leaves, while a small amount of callus was visible at the nodal base (Figure 1c). In the medium supplemented with thidiazuron (TDZ), almost of all the single node segments only developed two new axillary shoots with yellowish-green and compact callus at the base (Figure 1d); after a period of culture, these callus have the ability to differentiate into shoots. When the culture medium contained KT or zeatin (ZT), one node segment proliferated 8.3–11.6 into multiple shoots in 30 days, and no callus was induced (Figure 1e). However, the hyperhydricity of shoots was observed in the medium with kinetin (KT) (Figure 1f). BA and ZT emerged as the most productive with regard to the number of shoots per explant as opposed to all the cytokinins supplied at the same concentration (Table 1). In addition, these axillary shoots induced by BA were stronger and thicker than those other cytokinin-induced shoots.

### 2.2. Impact of PGRs on Shoot Proliferation from Leaf

**Figure 2:**
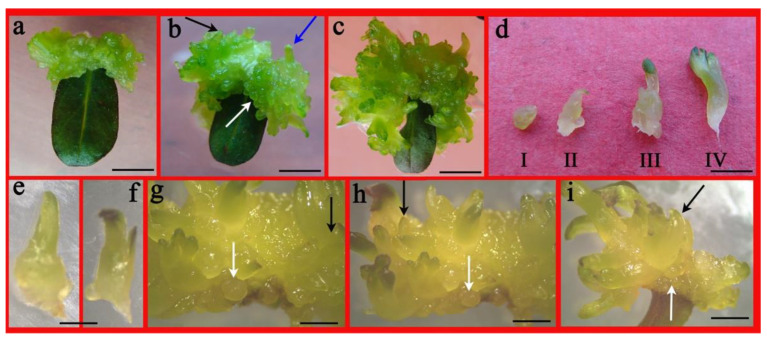
In vitro shoot regeneration and embryo-like structures (ELSs) induction from *A. lupulina* leaf explants on MS medium supplemented with 3.0 mg L^−1^ of TDZ and 0.1 mg L^−1^ of NAA. (**a**) Formation of callus following a 3-week culture. (**b**) Appearance of adventitious shoots (blue arrow) and some ELSs (in globular (white arrow) and torpedo shapes (black arrow)) on the leaf explant surface following culture for 4 weeks. (**c**) prolific adventitious shoots following 5 weeks of culture. (**d**) ELSs stages, globular- (I), torpedo-(II) and cotyledonary embryo (III,IV). Torpedo-(**e**) and cotyledon- (**f**) shaped embryo. (**g**–**i**) were amplified image of Figure 2b from different angles, the white arrow is indicative of ELSs globular-shaped embryo, the black arrow is indicative of torpedo-shaped embryo. Bar = 5 mm (**a**), 3 mm (**b**,**c**), 5 mm (**d**), 2 mm (**e**,**f**).

This experiment was performed by employing leaf explants on MS medium with TDZ and BA individually or with NAA. The addition of NAA augmented adventitious shoot regeneration and ELSs generation in *A. lupulina*. For 3.0–4.0 mg L^−1^ TDZ, one-third of the leaf explants turned brown (Table 2), with enlarged explants and ample callus initiated at wounded site surfaces (Figure 2a), except in the control following 3 weeks of culture. A higher level of TDZ (4.0 mg L^−1)^ did not significantly increase adventitious shoots and ELSs. The combination of (4.0 mg L^−1^) TDZ and NAA (0.1 mg L^−1^) augmented the adventitious shoots (26.4/leaf explant) as opposed to only TDZ in the medium (12.0/leaf explant), with the response at 81.6%. After 5 weeks, the greenish and compact callus almost covered the explants with some adventitious shoots (Figure 2b blue arrow) and globular-(Figure 2b,g–i white arrow; Figure 2d-I) and torpedo-shaped (Figure 2b,g–i black arrow; Figure 2d-II,e) somatic embryo-like structures evident on the callus surface. Extending the culture time by 2 weeks increased the visibility of more adventitious shoots with the emergence of a cotyledon-stage embryo (Figure 2d-III,IV,f) from the callus surface, which subsequently developed into adventitious shoots (Figure 2c).

**Figure 3:**
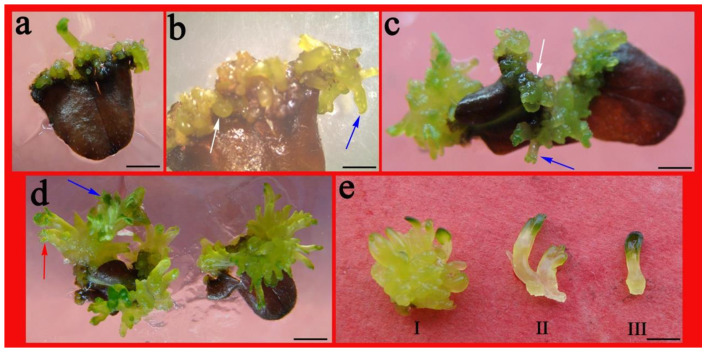
In vitro shoot regeneration and ELSs induction from *A. lupulina* leaf explants on MS medium plus 3.0 mg L^−1^ BA and 0.1 mg L^−1^ NAA. (**a**) Light-green callus formation at the edge of the browned blade following 3 weeks of culture. (**b**) Appearance of adventitious shoots (blue arrow) and some globular- shaped (white arrow) somatic embryos on the leaf explant surface following a 4-week culture. (**c**) Adventitious shoot (blue arrow) development and globular-shaped (white arrow) ELSs. (**d**) cotyledon-shaped (red arrow) formed and then developed into real shoots (blue arrow) after culture for 5 weeks. (**e**) Torpedo-shaped (I) and cotyledonary embryo (II,III) individual. Bar = 5 mm (**a**), 2 mm (**b**), 1 mm (**c**–**e**).

The browning rate was conspicuously more for either BA alone or with 0.1 mgL^−1^ NAA, (between 70.2 to 75.4%) as opposed to TDZ treatment only (ranged from 25.5–32.5%). Interestingly, the leaf explants turned brown but displayed no necrosis and sustained high regeneration capacity. The development of ELSs and enumeration of regenerated shoots were documented. The numbers of adventitious shoots and ELSs were more for the BA (4.0 mg L^−1^) and NAA (0.1 mg L^−1^) combination at 13.5 and 8.7, respectively, versus only BA (7.8 shoots and 4.7 somatic embryos, respectively) (Table 2). A light-green callus was induced on the cut surface of the enlarged and browning leaf in 3 weeks (Figure 3a). Later, the formation of few shoots (Figure 3b blue arrow) and a protuberance (globular-shaped, Figure 3b, white arrow) were detected on the callus in the subsequent culture period. Extending the culture time resulted in the formation of adventitious shoots (Figure 3c blue arrow) on the callus surface and some globular- (Figure 3c white arrow; Figure 3e-I) and cotyledon-shaped (Figure 3d red arrow; Figure 3e-II,III) ELSs were documented (Figure 3c–e) that later developed into real shoots (Figure 3d blue arrow).

### 2.3. Impact of PGRs on Adventitious Shoot Regeneration from Shoot Tips

**Figure 4:**
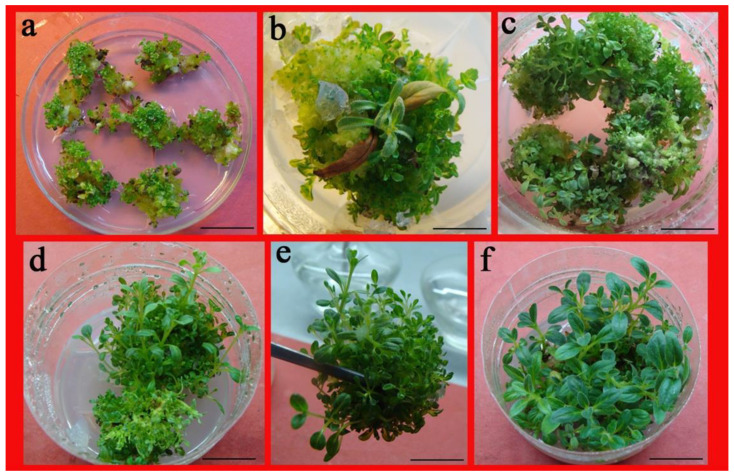
Callus-mediated plant regeneration from *A. lupulina* shoot tip explants on medium inclusive of 2.0 mg L^−1^ BA and 0.01 mg L^−1^ NAA. (**a**) Callus differentiation and bud initiation from shoot tip (Chopped) tissue for 3 weeks; (**b**,**c**) Prolific adventitious shoot development from light-green callus for 5 weeks. (**d**,**e**), Increasingly proliferated and differentiated shoot for 6 weeks. (**f**) Cultures grown for 7 weeks with robust shoots ready for rooting. Bar = 2 cm (**a**,**c**–**f**), 2.5 cm (**b**).

The chopped shoot tip explants yielded a yellowish-green, friable callus with the development of copious numbers of adventitious shoots from the callus following 21 days of culture on the same media (Figure 4a). After 35 d of culture, the shoots were trimmed with the division of the multiple shoot clumps divided into many smaller clumps (10–15 shoots/clump) for multiplication culture (Figure 4b,c). Three subcultures resulted in the production of numerous shoots (Figure 4d,e). Protracting the culture period resulted in robust, healthy prolific shoots for rooting (Figure 4f). The propagation coefficient was 25.4 on the 2.0-mg L^−1^ TDZ- and 0.01-mg L^−1^ NAA-containing medium, while, for the 2.0-mg L^−1^ BA- and 0.01-mg L^−1^ NAA-containing medium, the propagation coefficient was 14.5. This was indicative of a high propagation coefficient for both the media with 100% callogenesis (Figure 4a; Table 3).

### 2.4. Root Formation in Regenerated Shoots

**Figure 5:**
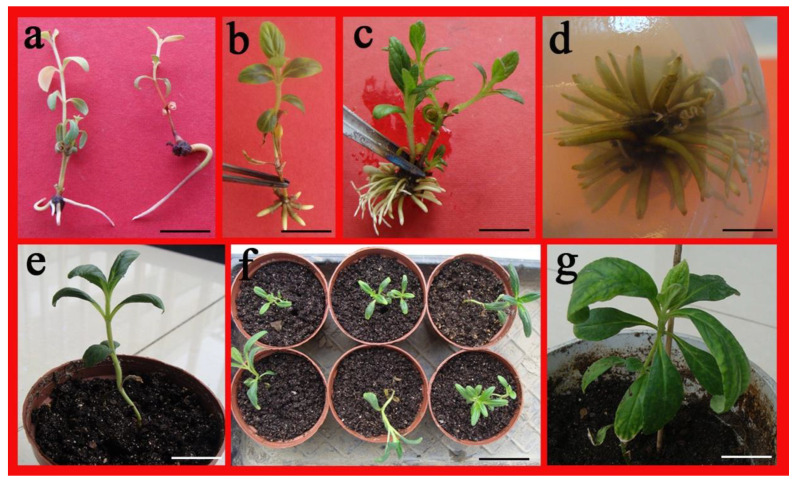
Rooting and transplanting of *A. lupulina*. (**a**–**d**) in vitro root production on 1.0 mg L^−1^ of IBA (**a**), 0.5 mg L^−1^ of NAA (**b**) and 1.5 mg L^−1^ of IBA plus 0.5 mg L^−1^ of NAA and activated charcoal (2 g L^−1^) (**c**,**d**). (**e**,**f**) acclimatization of a rooted plantlet in the culture room. (**g**) Completely regenerated plantlets grown in greenhouse. Bar = 1 cm (**a**,**b**,**c**), 5 mm (**d**), 2.5 cm (**e**), 1.5 cm (**f**), 3.0 cm (**g**).

Following the excision of healthy individual shoots of *A. lupulina* from multiplication, the half-strength MS medium was cultured and various concentrations of auxins were added. However, there were no roots formed on the 0.5-mg L^−1^ IBA or 0.1-mg L^−1^ NAA-supplemented culture. As opposed to single auxins, IBA in combination with NAA emerged as a strong root inducer for *A. lupulina*. The root numbers per shoot were between 3.1 and 25.8 except in controls (Table 4). The maximum root yield (25.8 ± 0.6) was observed in the 1.5-mg L^−1^ IBA- and 0.5-mg L^−1^ NAA-containing cultures in 3 weeks with the maximum rooting frequency up to 81.71% (Table 4). Only 32.5% of shoots rooted on 1.0 mg L^−1^ of IBA ex vitro with a few roots visible at the basal end of shoots (Figure 5a). Compared to other treatments, root development with lowered browning and more white roots was seen on the medium containing only 1.0 mg L^−1^ or 1.5 mg L^−1^ IBA alone or with 0.5 mg L^−1^ NAA. The mix of 1.5 mg L^−1^ of IBA plus 0.5 mg L^−1^ of NAA and activated charcoal (2 g L^−1^) (Figure 5c,d; white roots) emerged as being more effective for rhizogenesis than treatment with auxin alone (Figure 5b; green roots) in this study.

### 2.5. Acclimatization of Regenerated Plants

Successful hardening and acclimatization of the well-rooted plantlets were performed at a maximum in vitro survival of 75%. Following 2 weeks of hardening, transfer of the plantlets was carried out to greenhouse conditions with survival and regular growth of more than 80% of the plantlets (Figure 5e,f). The successful adaption was indicated by the appearance of new leaves from the ex vitro rooted shoots of these in vitro developed plantlets (Figure 5g).

## 3. Discussion

### 3.1. Axillary Shoot Proliferation

In vitro propagation via the multiplication of axillary shoots is an efficient system for mass producing true-to-type plants [14]. This approach sees the widespread usage of nodal segment explants as reported for *Portulaca pilosa* [15], *Caryopteris terniflora* [16], and *Teucrium scorodonia* [17]. Results along the similar line have been reported for the genus *Ajuga*. For instance, Sivanesan and Park [18] showed the importance of adding PGRs for axillary shoot proliferation from *A. multiflora* nodal explants; in particular, the effectiveness of BA was reported. Here, MS medium that lacked PGRs displayed no shoots. Four cytokinins successfully induced shoot regeneration and the shoot regeneration capacity decreased by increasing the concentration of cytokinins above the optimum level. BA was the most effective among the four cytokinins employed in axillary shoot regeneration in *A. lupulina*similar to that reported by Sivanesan and Park [18]. TDZ is believed to be the best synthetic cytokinin present for the regeneration of numerous plant systems [19,20]. High cytokinin activity of TDZ inhibits shoot elongation; however, it is conducive to the formation of somatic embryos [15,21]. Whereas, in our study, TDZ induced the fewest shoots with bulk differentiated callus supporting this view. Hyperhydricity exhibited in regenerated shoots of *A. lupulina* from KT at 2.0–4.0 mg L^−1^, while the other cytokinins did not. Hyperhydricity has been reported to be influenced by cytokinin concentration and type [22,23], which is consistent with our present findings. As shown in Table 1, some callus appeared at the base of nodal segment in the medium containing BA and TDZ, and callus was necessary for the induction of ELSs. Therefore, BA and TDZ would be used in subsequent experiments.

### 3.2. Embryo-Like Structures (ELSs)

Most micropropagation approaches for *Ajuga* have employed leaf explants, which appear to have better outcomes than other explants, such as petiole, root, and so on [2,3,11,12,13]. The literature is scarce regarding the somatic embryogenesis in *Ajuga* species except for *A. bracteosa*. In recent years, Gul [3] was the first to report somatic embryogenesis from A. bracteosa leaf explants. In our study, we also reported morphologically similar embryo-like structures (ELSs) induction from the leaf explants. The induction of adventitious shoots and ELSs have been simultaneously induced from explants of *Alupulina* that has also been found in other species as well, such as Portulaca Pilosa [15], *Metabriggsia ovalifolia* [21], and *Camellia nitidissima* [24]. This induction of adventitious shoots or somatic embryo-like shoots was seen on MS medium containing cytokinins (3.0–4.0 mg L^−1^ BA and TDZ) or along with 0.1 mg L^−1^ NAA (Figure 2c,i). TDZ and NAA were found as the best with regard to the mean numbers of shoots and somatic embryo-like structures as opposed to BA plus NAA, with the presence of NAA dramatically augmenting shoot proliferation and embryo numbers. The addition of auxin NAA was obviously beneficial to the formation of somatic embryo-like structures; these results are on the same lines for *Ajuga bracteosa* [12], *Sida cordifolia* [25] and *Lycium barbarum* [26]. The elimination of browning associated with in vitro culture is vital for successfully establishing cultures [24]. The PGR with the highest efficiency is TDZ, a phenyl urea derivative considered as the current number-one-ranking synthetic cytokinin for regeneration and somatic embryogenesis [27,28]. Our observations are in line with high-cytokinin emerging species beneficial for the somatic embryogenesis induction [21,24]. However, long exposure of high concentration TDZ resulted in browning, deformation of seedlings, necrosis, and, subsequently, death [20,29]. However, here, we found leaves containing BA brown but not TDZ, which may be due to different responses of different species to different cytokinins. Interestingly, the browning did not abolish the regeneration capacity of the leaf that showed callus induction to eventually develop into ELSs and shoots with values at 8.7 and 12.0 (Table 2; Figure 2 and Figure 3), respectively. Further, globular-, torpedo-, and cotyledon-shaped structures were observed, that developed into real shoots (Figure 3). Unfortunately, we did not observe SELSs germinated with distinct root system. The founding of approaches for somatic embryogenesis for in vitro mass propagating techniques and for conserving medicinally significant and endangered plants emerge as a vital and promising offshoot protocol [30].

### 3.3. Rooting

The effectiveness of NAA and IBA in root induction is reported in many species, including *Populus alba × P. glandulosa) × P. tomentosa* [31], *Jatropha curcas* [32], and *Ranunculus wallichianus* [33]. In our experiment, we found it difficult to root for *A. lupulina*. No free PGRs or low auxin concentration failed to induce root forming. In cases where IBA or NAA were used for rooting, the root frequency and the number of roots increased with increasing the auxin concentration; however, above the optimum level, they decreased. In addition, the effectiveness of root induction in the presence of IBA and NAA were relatively high, which is consistent with the findings in other species [34]. This suggests that the endogenous concentration of auxins in the shoots were not sufficient for root induction, as it was earlier reported by Piatczak and Wysokinska [35]. The addition of activated carbon is beneficial to rooting, and this conclusion has been reported in some species [33,36,37]. Here, we found that the base of the shoot was easily browned, and the addition of activated carbon effectively improved the rooting rate, which may be due to the adsorption of phenolic substances by activated carbon.

### 3.4. Optimal Explants

To date, micro-propagation of *Ajuga* species from leaf, petioles, shoot tips, nodal segment, and roots has only been reported for some species, excluding *A. Lupulina* species [2,3,11,12,13]. Leaf is often the first choice for tissue culture because of its convenience and abundance of raw materials. More important is that leaf, i.e., the basic material of genetic transformation [38,39]. Three kinds of explants, namely nodal segment, leaf, and shoot tip, were used in our study. Shoot regeneration was successfully induced from all three types of explants from *A. Lupulina* by two pathways (direct (via callus) and indirect organogenesis (not via callus)). Significant differences in regeneration were observed among the three explant types (leaf > shoot tip > nodal segment). The callus induced by leaf is the basis for further genetic transformation of *A. Lupulina*.

The optimum concentration and type of PGRs is different for different explants [16,31]. In our results, for nodal segment, the most optimal cytokinin is 4.0 mg L^−1^ of BA (10.2/explant), and the shoot are robust. Furthermore, 4.0 mg L^−1^ of TDZ, in combination with 0.1 mg L^−1^ of NAA (26.4 shoot and 12 ELSs/explants), was suitable for leaf explants. NAA 0.01 and TDZ 2.0 (25.4/explant) were the best media for shoot tip induction. Similar phenomena were reported in *Caryopteris terniflora* [16], (*Populus alba* *× P. glandulosa*) × *P. tomentosa* [31].

## 4. Materials and Methods

### 4.1. Plant Source and Culture Details

The field-grown plants of *Ajuga lupulina* were collected from Balang Mountain, Wolong zone (30°51′28″–30°53′6″ N, 102°55′14″–102°58′42″ E at an altitude of 3800 m), in the Eastern Sichuan Province of China. They were maintained under glasshouse conditions and were the source of plant material. These plants were previously identified it by Professor Luo Minghua of Mianyang Normal University. Young nodal segments were used as explants for primary culture. The plant segments were sterilized by running tap water for 30 min, followed by 30 s in 70% ethanol, 4 min in 0.1% mercuric chloride, and a final wash in sterile water. Once sterilized, the plant segments were placed on MS medium containing different plant growth regulators (PGRs). MS basal medium was gelled with 3% (*w*/*v*) sucrose, and the pH was set to 5.7 before adding 0.7% (*w*/*v*) ager (Sigma-Aldrich, St. Louis, MO, USA) and autoclaving at 121 °C for 15 min. The cylindrical culture flask was 7.0 cm (diameter) and 8.0 cm (height) while Petri dishes employed were 9 cm in diameter. All assays were inclusive of a 12-h photoperiod with a light intensity of 80 µmol m^−2^ s^−1^ and a constant temperature of 25 ± 2 °C. Four nodal segments were inoculated in each culture flask to induce new axillary shoots, which were sub-cultured fortnightly. After a month of subculture, multiple shoots and broad leaves were obtained suitable for the assays mentioned below. The PGRs used in the experiments were auxin including naphthalene acetic acid (NAA), indole-3-butyric acid (IBA), cytokinin 6-benzyladenine (BA), thidiazuron (TDZ), zeatin (ZT), and kinetin (KT).

### 4.2. Effect of PGRs on Leaf-Induced Adventitious Shoots and Somatic Embryo-Analogy like Structures (ELSs)

In vitro inoculation of single leaves with the adaxial side down onto MS medium containing various PGRs and their combinations for inducing adventitious shoots and ELSs was performed, with the control as MS medium minus PGRs. There were 10 Petri dishes with 30 explants in duplicate assays each. Following 5 weeks of culture, shoot regeneration and ELSs development were assessed and compared.

### 4.3. Effect of PGRs on Shoot Tip Adventitious Shoot Regeneration

Shoot tips (chopped) were cultured in MS medium with 2.0 mg L^−1^ of BA or TDZ with 0.1 mg L^−1^ of NAA for inducing the callus and shoot regeneration. The samples with high differentiation ability were divided into several smaller clumps for multiplication culture. After four subcultures on respective media, quantification of the propagation coefficient per subculture was carried out as follows: (number of shoots following inoculation)/(number of shoots prior to inoculation) followed by quantification of the mean propagation coefficient of the four subcultures. Each treatment contained 10 culture flasks with 30 explants. The experiments were repeated three times. After 6 weeks, the data on callogenesis and the shoot proliferation coefficient were recorded.

### 4.4. Root Formation, Acclimation and Transplantation

Following the excision of single shoots (3–4 cm long) with four to five leaves from mass cultures, transfer was achieved to half-strength MS medium followed by treatment with auxins at varying levels and combinations for the induction of roots, and 0.2% active charcoal with each treatment having 30 (3 × 10) shoots. The control was medium lacking active charcoal or auxins. After 3 and 5 weeks of culture, the browning rate, induction, and root formation were assayed. Removal of healthy plantlets (5–7 cm in height) was completed after a total of 8 weeks, followed by gentle water rinsing to remove the agar. Eighty plantlets were transplanted into plastic pots (8.4 cm in diameter × 7.2 cm high) containing peat, perlite, and vermiculite (1:1:1, *v*/*v*/*v*). Transparent plastic cups were employed to cover the pots to maintain moisture over 2 weeks of greenhouse acclimatization which entailed the gradual removal of these cups. The greenhouse temperature was maintained at 25 ± 2 °C with a light intensity of 80 µmol m^−2^ s^−1^, the humidity was 70%, and plants were watered daily and supplied with Hoagland nutrient solution every five days. Plant survival rate (%) was assessed on the 30th day after transplantation.

### 4.5. Statistical Analysis

Analysis entailed the usage of one-way analysis of variance (ANOVA) with the differences with controls deemed as statistically significant by the least significant difference (LSD) test at *p* ≤ 0.05 employing SPSS 18.0” (IBM, Armonk, NY, USA).

## 5. Conclusions

In this work, we established protocols for direct and indirect regeneration of *A. lupulina* and embryo-like structures (ELSs). The explant type and cytokinin combinations significantly influenced *A. lupulina* regeneration. While TDZ promoted ELSs development from leaf explants, BA favored shoot regeneration from nodal segment explants for rapid mass clonal propagation. The direct or indirect regeneration system described here can potentially be used for multiplication. It can also provide an approach for genetic transformation. Additionally, ELSs are prospective material for artificial seed preservation of this vital species.

## Figures and Tables

**Table 1:** Effect of cytokinins on axillary shoot proliferation from nodal segments of *A. lupulina* after culture for 35d.

Cytokinin (mg L^−1^)	Axillary Shoot Proliferation (Per Explant)	Visible Appearance		
		The Color of the Leaves	Callus	Shoot Growth State
Control	0	Nil	Nil	Nil
BA 2.0	7.7 ± 0.5 d	Dark-green;	a small amount;	+++
BA 3.0	10.2 ± 0.1 ab	Dark-green;	a small amount;	++++
BA 4.0	9.0 ± 0.3 b	Dark-green;	a small amount;	+++
TDZ 2.0	1.5 ± 0.1 e	Dark-green;	a large amount;	+++
TDZ 3.0	2.2 ± 0.2 e	Dark-green;	a large amount;	+++
TDZ 4.0	1.8 ± 0.3 e	Dark-green;	a large amount;	+++
ZT 2.0	8.3 ± 0.5 c	Light-green;	no callus;	++
ZT 3.0	9.6 ± 0.3 b	Light-green;	no callus;	++
ZT 4.0	9.4 ± 0.4 b	Light-green;	no callus;	
KT 2.0	9.1 ± 0.7 b	Light-green;	no callus;	+Hyperhydricity
KT 3.0	11.6 ± 0.3 a	Light-green;	no callus;	+Hyperhydricity
KT 4.0	10.4 ± 0.2 ab	Light-green;	no callus;	+Hyperhydricity

Data are presented as means ± standard deviations from three replicates with 30 explants in each replicate. Different letters within a column indicate significant differences according to Duncan’s multiple range test (*p* < 0.05). “+” indicates shoot growth condition, and the more +, the better growth condition.

**Table 2:** Effect of PGRs on induced morphogenesis from leaf explants of *A.lupulina*.

Treatment (mg L^−1^)	Browned (%)	Response (%)	Shoots Number	ELSs Number	In Vitro Morphogenesis
BA 3.0	75.4 ± 2.4 b	43.1 ± 2.2 d	5.5 ± 1.4 e	3.3 ± 1.5 d	Leaf blade browned; adventitious shoot; globular somatic-analogy embryo (ELSs) buds
BA 4.0	77.2 ± 3.6 b	47.5 ± 3.2 cd	7.8 ± 2.3 e	4.7 ± 1.4 d	Leaf blade browned; adventitious shoot; globular ELSs buds
BA 3.0 + NAA 0.1	72.5 ± 2.4 b	54.2 ± 2.1 c	12.4 ± 0.9 d	7.6 ± 0.7 c	Leaf blade browned; adventitious shoot; globular ELSs buds
BA 4.0 + NAA 0.1	70.2 ± 3.1 b	57.6 ± 1.9 c	13.5 ± 3.2 d	8.7 ± 1.4 b	Leaf blade browned; adventitious shoot; globular ELSs buds
TDZ 3.0	32.5 ± 2.5 c	72.4 ± 2.5 b	17.5 ± 5.6 c	5.3 ± 1.2 c	Leaves darkened; adventitious shoot; globular ELSs buds
TDZ 4.0	35.7 ± 3.2 c	79.3 ± 3.4 b	20.8 ± 2.1 bc	6.3 ± 0.8 c	Leaves darkened; adventitious shoot; globular ELSs buds
TDZ 3.0 + NAA 0.1	25.5 ± d	83.5 ± 3.2 a	22.5 ± 3.2 ab	9.3 ± 1.4 b	Leaves darkened; adventitious shoot; globular ELSs buds
TDZ 4.0 + NAA 0.1	27.8 ± d	81.6 ± 3.5 ab	26.4 ± 2.1 a	12.0 ± 2.2 a	Leaves darkened; adventitious shoot; globular ELSs buds

Data are presented as means ± standard deviations from three replicates with 30 explants in each replicate. Different letters within a column indicate significant differences according to Duncan’s multiple range test (*p* < 0.05).

**Table 3:** Effect of PGRs on induced organogenesis from shoot tip of *A. lupulina*.

Treatment (mg L^−1^)	Callogenesis (%)	Shoot Proliferation Coefficient	Observed Results
Control	0 b	0 c	Nil
NAA 0.01 + TDZ 2.0	100 a	25.4 ± 2.2 a	Light-green callus; Soft and fragile; Dwarf shoot
NAA 0.01 + 6-BA 2.0	100 a	18.5 ± 3.7 b	Light-green callus; Soft and fragile; Robust shoot

Data are presented as means ± standard deviations from three replicates with 30 explants in each replicate. Different letters within a column indicate significant differences according to Duncan’s multiple range test (*p* < 0.05).

**Table 4:** Effects of auxins on root induction in *A. lupulina*.

Treatment (mg L^−1^)	Culture Time		Browned (%)	Rooting (%)	Mean Number of Shoots with Roots	Morphology
	3 Weeks	5 Weeks				
0	−	−	88.4 ± 3.4 a	0 e	0 d	—
IBA 0.5	−	−	75.4 ± 4.1 b	0 e	0 e	White
IBA 1.0	+	+	52.8 ± 2.2 d	32.5 ± 1.2 c	3.1 ± 0.3 d	White
IBA 1.5	+	+	54.6 ± 2.3 d	40.6 ± 1.4 c	5.3 ± 0.5 c	White
IBA 2.0	+	+	58.8 ± 2.4 d	35.3 ± 1.1 c	3.3 ± 0.7 d	White
NAA 0.1	−	−	77.7 ± 2.6 b	0 e	0 d	Green
NAA 0.5	−	+	68.3 ± 3.2 c	22.5 ± 1.8 d	5.4 ± 0.1 c	Green
NAA1.0	−	+	65.2 ± 0.2 c	17.4 ± 1.3 d	3.5 ± 0.2 d	Green
IBA 1.0 + NAA 0.5	+	+	32.5 ± 1.7 e	70.1 ± 2.1 b	14.6 ± 0.2 b	White
IBA 1.5 + NAA 0.5	+	+	35.7 ± 2.3 e	81.71 ± 2.4 a	25.8 ± 0.6 a	White

Data are presented as means ± standard deviations from three replicates with 30 explants in each replicate. Different letters within a column indicate significant differences according to Duncan’s multiple range test (*p* < 0.05). All treatments included 1/2 MS medium. + indicates root formation, and—indicates roots were not formed yet within the observed time period.

## Data Availability

All data generated or analyzed during this study are included in this published article.
